# Research on a floating thermoelectric power generator for use in wetland monitoring

**DOI:** 10.1371/journal.pone.0232331

**Published:** 2020-05-05

**Authors:** Yuqi Zhang, Zhe Zhang, Yafeng Wu, Latai Ga, Daochun Xu, Wenbin Li

**Affiliations:** 1 School of Technology, Beijing Forestry University, Beijing, China; 2 Key Lab of State Forestry Administration on Forestry Equipment and Automation, Beijing, China; Universita degli Studi della Tuscia, ITALY

## Abstract

A floating power generation device is designed and fabricated to overcome the power supply limitations of wireless sensor networks for environmental monitoring. Once there is a temperature difference between the upper surface exposed to sunlight and the lower surface in the water, the device is capable of generating power while floating in the wetland environment. Fresnel lenses were applied to concentrate solar irradiation on a selective absorbing coat. Meanwhile two vertical axis rotors were used to cool the cold side of the thermoelectric power generator by catching the breeze. The effects of solar irradiation, temperature distribution, load resistance, wind speed, the maximum power and the electrical efficiency of the thermoelectric power generator were analyzed. When subjected to solar irradiation of 896.38 W/m^2^, the device generated a potential difference of 381.03 mV and a power output of 8.86 mW via thermoelectric generation. In addition, compared with the system without wind, the output power was increased by approximately 10.96% in our system. The low power wireless networks, used in wetland environments, could be operated by the thermoelectric power generated by the floating device. Besides, this system offers powering solution for self-power miniature devices that are applied in aqueous environment.

## Introduction

Meeting future energy demands using renewable and green technologies is a significant global challenge [1–3]. The replacement of fossil fuels with sustainable energy sources has attracted increasing interest in recent years. While thermal energy is the most potential alternative because of higher efficiency and lower costs [4]. In recent years, Thermoelectricity has been the core of green energy and sustainable energy application which mainly focus on energy sources, energy transmission and thermodynamic materials conversion. [5]. Waste heat or solar energy could be directly converted into electricity using thermoelectric generator devices that utilize the Seebeck effect [6].

So far, a mass of studies on thermoelectric generator (TEG) have been carried out because of its many advantages such as no noise, no extra waste and low cost and so on. While they mainly focuses on the photovoltaic-thermoelectric hybrid systems [7–9], wearable thermoelectric generators [10], and the power supply for underwater gliders [11] and wireless sensors [12, 13]. Although TEG have the potential to be used in a variety of areas and to supply electrical power for low-power components, the relatively low conversion efficiency has become its primary limitation.

In order to solve this problem, the optical concentrator and the cooling systems have been introduced to maintain the temperature difference across a thermoelectric generator. Concentrating Fresnel lenses are widely used in solar cell [14] sand thermoelectric generators [9, 15–17] that make use of optical concentrator systems. Willars et al [9] investigated a solar hybrid system with Fresnel lens, of which the hot side temperature succeed in reaching 437K. Besides, Nia et al [16] proposed a cogeneration solar system in which Fresnel lens can transfer heat to TEG by the intermediate fluid, and the maximum temperature is 91.2°C. The high temperature of hot side can be maintained by the use of Fresnel lens.

In addition, the performance of thermoelectric device is determined by its heat exchangers [18]. Lv et al [19] and Li [20] studied the fluid cooling exchangers by changing the mass flow rates of water and air. The results showed that the temperature difference increased firstly and then kept stable, with the increase of water and wind speed for cooling system. The above are mainly concentrated on the choice of stable flow rates for fluid cooling exchangers. However, the mass flow rates of water and air are intermittent, transient and unpredictable in nature. The normal heat exchangers of TEG are not suitable for wireless sensors network (WSN), which are increasingly used for monitoring complex aqueous environment. Lee et al [21, 22] proposed a floating energy harvester using TEG, but the radiator limited the efficiency of this device. Therefore, a passive heat exchanger was proposed for self-power miniature devices in aqueous environments.

Approximately 71% of the earth’s surface is covered by water, and WSN can be used to measure and record a wide variety of information, including flow rate, temperature and water quality. The power supply of terrestrial wireless applications are easily maintained. However, it is extremely challenging, even impossible, to change batteries in drifting devices and stationary floating devices [21, 22]. The self-powered floating devices with TEG offers a potential solution to this problem. The power generated by the floating devices were sufficient to run self-powered miniature devices.

In this study, a floating thermoelectric power generation device that concentrates solar energy was designed and fabricated. The structure of the device had a considerable influence on the efficiency of electricity production. A floating cavity with TEG provided power to WSN by the application of Fresnel lens. The two vertical axis rotors were designed as the cooling system, which could catch the breeze to enhance the flow rates of cold side. Compared with alternative thermoelectric conversion devices, this device offers a number of advantages:

1. Solar irradiation was captured efficiently using Fresnel lenses and the cavity structure significantly reduced thermal radiation and thermal convection.
2. The power supply system was made of light-weight, waterproof materials which floated steadily on the water.
3. The small size of the device will allow it to be used in varieties of wetland environments, while providing power for wetland monitoring. The power output was fourfold that of another device with the same numbers of TEG modules.
4. The device used both breeze and water flow for a cooling system.

## Design of the floating device

### Device structure

**Fig 1:**
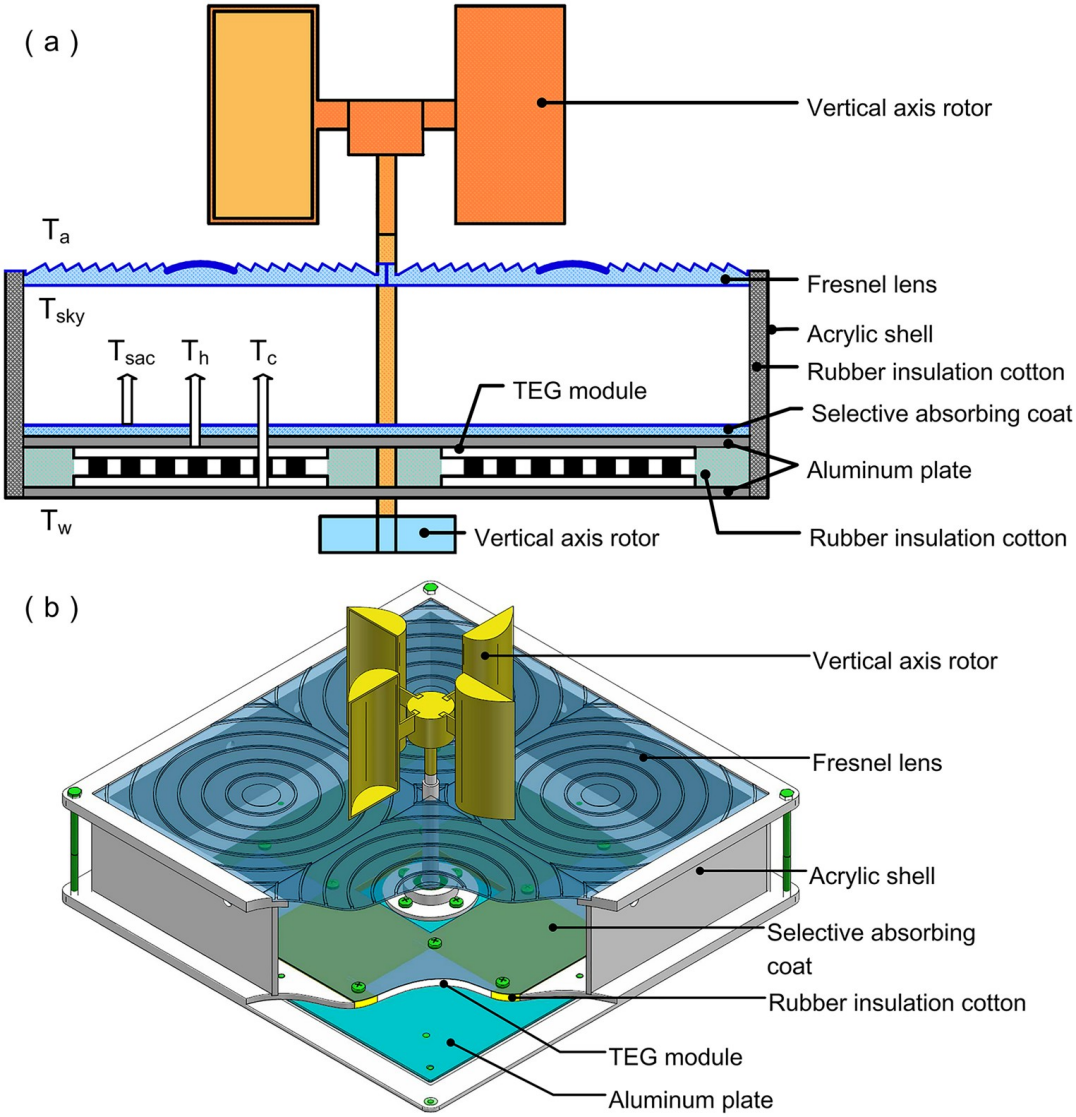
The floating device. (a) 2D schematic diagram of the device and (b) 3D schematic diagram of the device.

A 2D schematic diagram of the floating device is shown in Fig 1(a). The device consisted of an optical concentrator module, a solar absorber module, a thermoelectric power generating module, and a cooling exchanger module.

Fresnel lenses were used for the optical concentrator module. They concentrated the solar radiation on a focal point and enhanced the solar energy density on the aluminum plate that was covered by a selective absorption coating. This coating had a low reflection over the solar spectrum region and a high reflection on the infrared wavelength region.

Four thermoelectric power generators were connected in series, and were attached to the back side of the aluminum plate (Fig 1(a)). A TEG module was located at the focal point of the Fresnel lens. As shown in Fig 1(b), in order to ensure the transfer of efficient heat, the four bolts are used around the four corners of the TEG module.

The floating device used water as a coolant on the cold side of the TEG module. The second aluminum plate (1 mm thick) was attached to the cold side of the TEG module. It both dissipated heat and protected the TEG modules from being damaged by the water (Fig 1(a)). The cooling exchanger module included two vertical axis rotors that could operate under different wind directions. It harvest wind energy at very low (≤ 1 m/s) and very high (≥25 m/s) wind speeds. Therefore, these vertical axis rotors have great promise for use as a cooling system in complex aqueous environments. The two vertical axis rotors were connected via a shaft that ran in miniature bearings. When the fins caught the breeze, the lower rotor agitated the water, which helped lower the temperature of the cold side.

#### Thermal insulation used in the solar absorber and device sealing

Thermal insulation is another key factor for efficient power generation. An acrylic shell (50 mm high), covered by a thermally insulating material, was used to minimize heat loss when the solar irradiation was concentrated by the Fresnel lenses (Fig 1(a)). Additionally, thermal insulation (Luyikeilinsi Technology Co., Ltd, China) was used in the gap between the two aluminum plates to ensure the heat could only pass through the hot side of the TEG modules. A rubber-plastic insulating material was used for the thermal insulation. The thermal conductivity, thickness, density and operating temperature of the thermally insulation material was 0.034W/ (m· k), 5 mm, 45 kg/m^3^ and −50–120 °C, respectively. The device was sealed to ensure that water could not enter and destroy the TEG modules. Bolts were used to fix the aluminum plate to the acrylic shell and all the gaps were sealed using silicon sealant, as shown in Fig 1(b).

### Mathematical model

**Fig 2:**
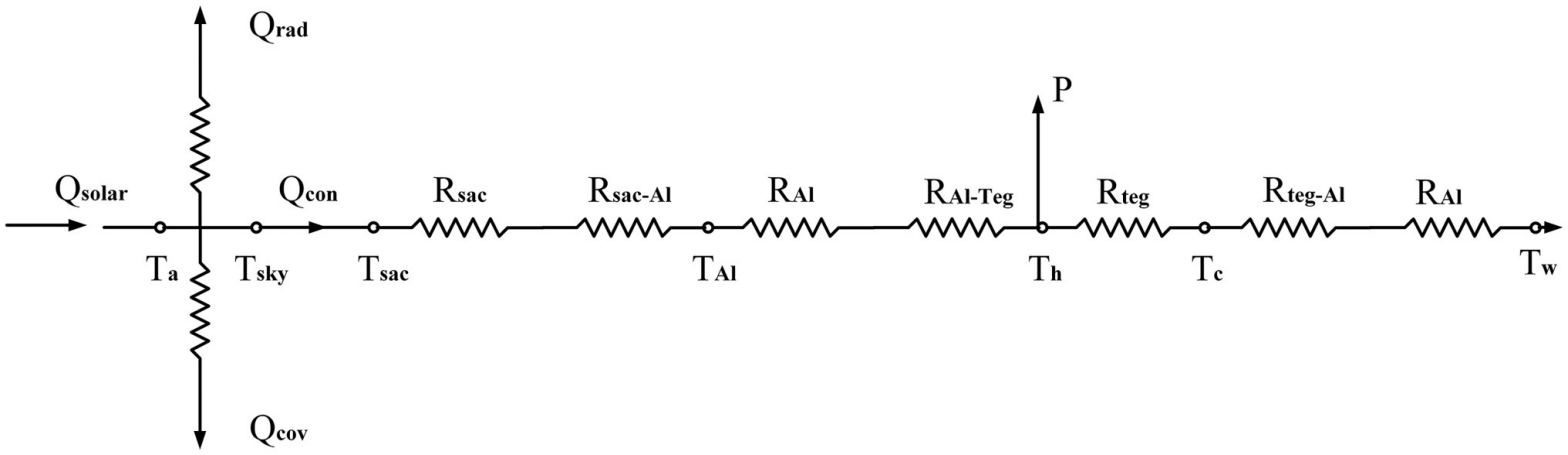
The heat transfer network of the floating device.

A thermodynamic analysis of this system was performed, which showed that minimizing the air flow above the hot side of TEG modules and increasing the temperature difference would improve the efficiency of the power generation system. The mathematical model included two parts: (1) the solar absorber module and (2) the TEG module. All equations were based on energy balance and heat transfer, as shown in Fig 2.

#### Solar absorber module

The solar absorber module consists of Fresnel lenses that concentrated solar irradiation on the selective absorbing plate. The heat concentrated on the selective absorbing coating can be expressed as follows [15]:

$$Q_{\text{in}}=Q_{con}+Q_{rad}+Q_{\text{cov}}$$ (1)

where *Q*_in_ is the solar energy absorbed by the selective absorbing coating. It can also be expressed as follows:

$$Q_{in}=\alpha_{b}CAG$$ (2)

$$C=A_{F}/A_{f}$$ (3)

where *α*_*b*_, *C*, *A*, *A*_*F*_, *A*_*f*_, and *G* are the solar absorbance, concentration ratio, area of the coating, area of the Fresnel lens, area of focal spot respectively and solar radiation, respectively.

*Q*_*rad*_ is the radiative heat transferred to the cavity. It is described as follows:

$$Q_{rad}=h_{rad}A(T_{sac}-T_{ct})$$ (4)

in which *h*_*rad*_ can be defined as:

$$h_{rad}=\varepsilon \delta (T_{ct}^{2}+T_{sac}^{2})(T_{ct}+T_{sac})$$ (5)

*Q*_*cov*_ is the heat convection from the selective absorbing plate to the square cavity, which is described as follows:

$$Q_{\text{cov}}=h_{\text{cov}}A(T_{sac}-T_{ct})$$ (6)

in which *h*_cov_ can be defined as [20]:

$$h_{\text{cov}}=6.5+3.3u$$ (7)

where *u* is the air flow rate within the square cavity. The low air flow rate decreases *Q*_cov_, which causes *Q*_con_ to increase.

*Q*_*con*_ is the energy conducted via the selective absorbing coating and can be expressed as follows:

$$Q_{con}=(T_{sac}-T_{h})/R_{sac-Al}$$ (8)

where *R*_*sac*−*Al*_ is the thermal resistance of the aluminum plate covered by the selective absorbing coating and *T*_*h*_ is the hot side temperature of the TEG modules.

#### TEG module

The TEG module converts thermal energy into electrical energy by a temperature gradient. The heat that passes through the hot side of the TEG module can be expressed as follows [15]:

$$Q_{tegh}=2n_{teg}\alpha_{teg}T_{h}I+2n_{teg}\frac{a_{teg}k_{teg}}{l_{teg}}(T_{h}-T_{c})-\frac{1}{2}I^{2}2n_{teg}\frac{r_{teg}l_{teg}}{a_{teg}}$$ (9)

$$Q_{tegl}=2n_{teg}\alpha_{teg}T_{c}I+2n_{teg}\frac{a_{teg}k_{teg}}{l_{teg}}(T_{h}-T_{c})+\frac{1}{2}I^{2}2n_{teg}\frac{r_{teg}l_{teg}}{a_{teg}}$$ (10)

The hot side absorbs the energy (*Q*_tegh_) from the aluminum plate that is covered by a selective coating, while the cold side releases the heat (*Q*_tegl_) through another aluminum plate attached to the cold side of TEG modules.

The electrical efficiency of the TEG module can be expressed as follows:

$$\eta_{teg}=\frac{Q_{tegh}-Q_{tegl}}{Q_{tegh}}$$ (11)

while the electrical efficiency of the floating device can be expressed as follows:

$$\eta_{sys}=\frac{Q_{tegh}-Q_{tegl}}{CAG}$$ (12)

The output power (*P*_*out*_) is a critical parameter that determines the performance of the power system. It is expressed using the following equation [20]:

$$P_{out}=\frac{\alpha_{teg}^{2}R_{load}}{{\left(R_{load}+r_{teg}\right)}^{2}}{\left(T_{h}-T_{c}\right)}^{2}$$ (13)

The maximum *P*_*out*_ can be obtained when the load resistance (*R*_*load*_) is equal to the internal resistance. The maximum *P*_*out*_ is expressed as follows:

$$P_{\text{max}}=\frac{\alpha_{teg}^{2}{\left(T_{h}-T_{c}\right)}^{2}}{18r_{teg}}$$ (14)

#### Heat exchanger

The heat that passes through the cold side of the TEG module can be also expressed as the heat flux via the aluminum plate to the water environment.

$$Q_{tegl}=\frac{T_{c}-T_{w}}{R_{teg-Al}+R_{Al}}$$ (15)

The thermal resistance of the aluminum plate *R*_*Al*_ includes two parts: the heat conduction in solid and the heat convection from the aluminum plat to the aqueous environment.

$$R_{A\text{l}}=R_{\text{con-}A\text{l}}+R_{\text{cov}-Al}$$ (16)

$$R_{con-Al}=\frac{H}{k_{Al}S_{1}}$$ (17)

$$R_{\text{cov}-Al}=\frac{1}{h_{\text{cov}-Al}S_{2}}$$ (18)

And the average heat transfer coefficient *h*_*cov*−*Al*_ is obtained from [21]

$$h_{\text{cov}-Al}=\frac{0.037L^{-\frac{1}{5}}\rho C_{\text{p}}u_{\infty }^{\frac{4}{5}}}{v^{-\frac{1}{5}}{\text{Pr}}^{\frac{2}{3}}}$$ (19)

Thus, the thermal resistance of the aluminum plate is

$$R_{Al}=\frac{H}{k_{Al}S_{1}}+\frac{v^{-\frac{1}{5}}{\text{Pr}}^{\frac{2}{3}}}{0.037L^{-\frac{1}{5}}\rho C_{\text{p}}u_{\infty }^{\frac{4}{5}}S_{2}}$$ (20)

## Materials and methods

**Fig 3:**
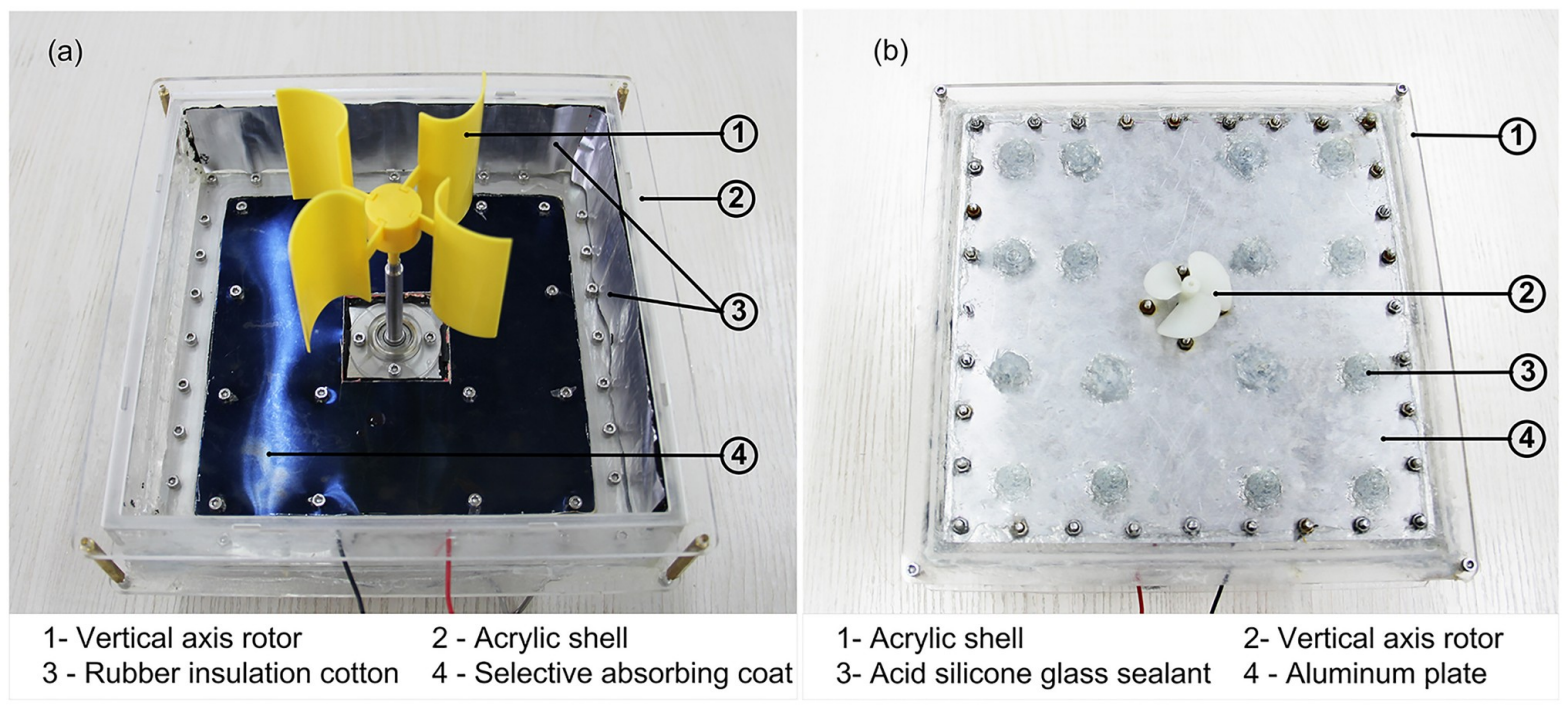
Photographs showing (a) the internal construction and (b) bottom of the floating device.

Photographs showing the internal construction and the bottom of the floating device are shown in Fig 3(a) and 3(b), respectively.

### Optical concentrator module and solar absorber module

The optical concentrator module contained Fresnel lens (Shandong Yuying Optical Instrument Co., Ltd.) made from acrylic with the area *A*_*F*_ = 0.1m × 0.1m × 4 = 0.04m^2^, a screw pitch of 0.3 mm, a luminousness of 92%, a thickness of 2 mm and the focal length of 50 mm.

The selective absorbing coat had a solar absorbance of 95% from 5–25μm and a thermal emittance of 5%. The thickness of the aluminum plate was 0.4 mm.

### Thermoelectric power generator module

The TEG module generated energy using the temperature differential between the hot and cold surfaces of the device, with water flow being used as a cooling technique.

A TG-127-08-04 thermoelectric power generator, which was produced by LEIZIH Thermoelectric Technologies Ltd., was used in the floating device. The size of the TEG module was 40 × 44 × 3mm^3^. The parameters of the TEG modules are given in Table 1.

**Table 1:** Parameters of TEG module.

Parameter	Numerical
Type	TG-127-08-04
Numbers of P or N junction *n*_*teg*_	127
Cross-section area of one P or N junction *a*_*teg*_	1.7 × 10^−6^m^2^
Electrical resistivity of one P or N junction *r*_*teg*_	1.45 × 10^−5^Ω/m
Seebeck coefficient *α*_*teg*_	2 × 10^−4^V/K
Thermal conductivity of TEG *k*_*teg*_	1.5W/K
Heat of TEG *l*_*teg*_	3 × 10^−3^m
Greatest variation *V*_*DC*_	7.71 V
Electrical current	3.6 A
Internal electrical resistance *R*_*TE*_	1.85 Ω
Maximum output power *P*_*MAX*_	7.5 W
Hot side temperature *T*_*hot*_	230 °C
Cold side temperature *T*_*cold*_	50 °C

## Results and discussion

**Fig 4:**
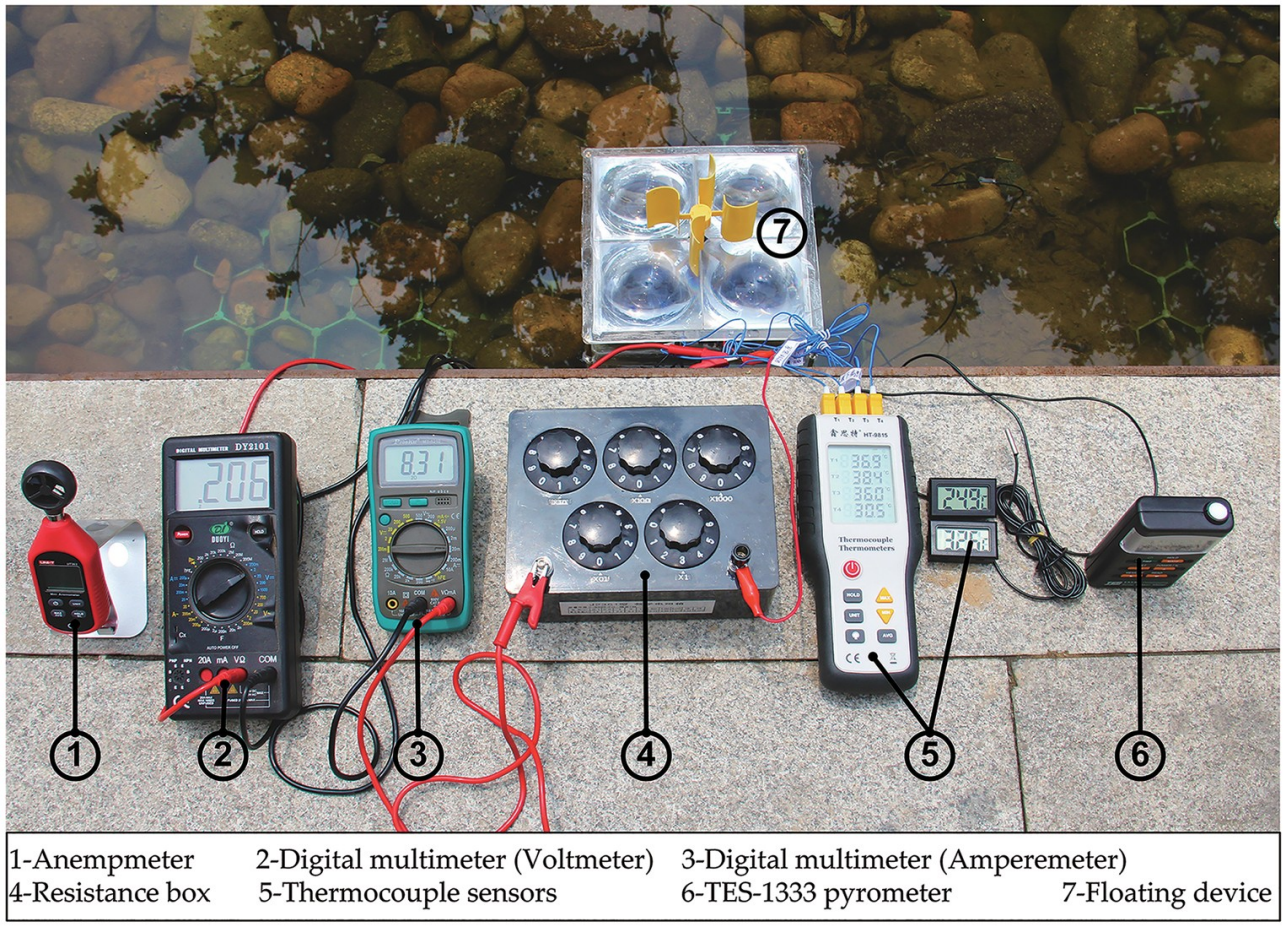
Photograph showing the floating device and the data measuring instruments.

Fig 4 shows the environment used for the experiments. The power system floated over the landscape pool (6m × 3m × 0.5m) in direct sunlight. The landscape pool was a miniature artificial wetland. There were no buildings or trees around to reduce the solar irradiation and wind speed. The experiment was performed in Beijing (39°54′*N*/116°28′E), Northern China on May 6th and lasted for six hours (10:00 to 16:00).

The efficiency of this system depended on several factors, including current, voltage, temperature, wind speed and solar irradiation. These parameters were recorded every 15 min using the instruments shown in Fig 4. A TES-1333 pyrometer was used to record the solar irradiation. The following six different temperatures were recorded (see Fig 1(a)): (1) the ambient temperature (*T*_*a*_), (2) the surface temperature of the selective absorbing coating (*T*_*sac*_), (3) the hot side temperature (*T*_*h*_), (4) the cold side temperature (*T*_*c*_), (5) the water temperature (*T*_*w*_), and (6) the cavity temperature (*T*_*ct*_). Two thermocouple sensors were attached to the hot and cold side of TEG modules and another sensor was attached to the surface of the selective absorbing coating. To ensure maximum power (*P*_*max*_), the four TEG modules were wired in series with a load resistor and an ammeter. Another digital multimeter recorded the voltage in parallel with the external resistor and the ammeter.

The output power of the floating device was correlated with the temperature difference, solar irradiance, voltage and wind speed. These parameters were recorded and discussed later.

**Fig 5:**
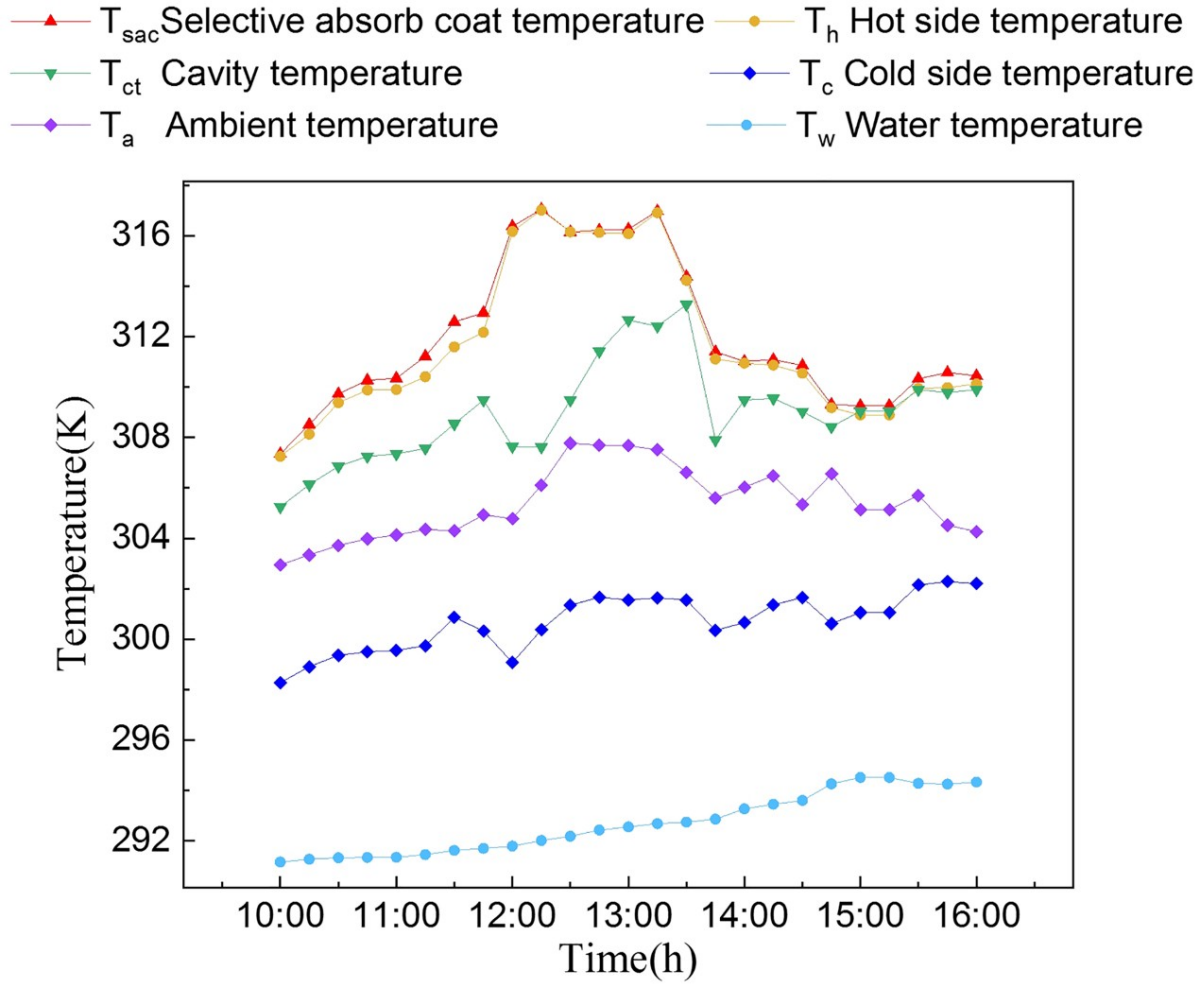
Six different temperatures (*T*_*sac*_, *T*_*h*_, *T*_*ct*_, *T*_*c*_, *T*_*a*_ and *T*_*w*_) were measured.

In addition to the semiconductor material, the temperature differential affects the conversion efficiency and power generation of the TEG modules. *T*_*a*_, *T*_*sac*_, *T*_*h*_, *T*_*c*_, *T*_*w*_ and *T*_*ct*_ are shown in Fig 5. The square cavity provided an environment with a stable temperature for the hot side of TEG modules. Both *T*_*sac*_ and *T*_*h*_ increased until noon because of enhanced irradiation, reaching a maximum at 12 pm. From 12 pm to 1:30 pm, *T*_*sac*_ and *T*_*h*_ were relatively stable at approximately 316.15 K, after which they began to decrease because of the decreasing solar irradiation. *T*_*a*_ and *T*_*c*_ followed the similar trend. The solar irradiation peaked at 1:30 pm, at which point *T*_*ct*_ reached a maximum of 313.15 K. The *T*_*ct*_ at the end of the experiment was the same as *T*_*h*_. *T*_*w*_ experienced a slight increase in temperature (294.15 K) at 4 pm. Both temperature of the cold side and water exhibited upward trends because the solar irradiation increased the surface temperature of the water.

**Fig 6:**
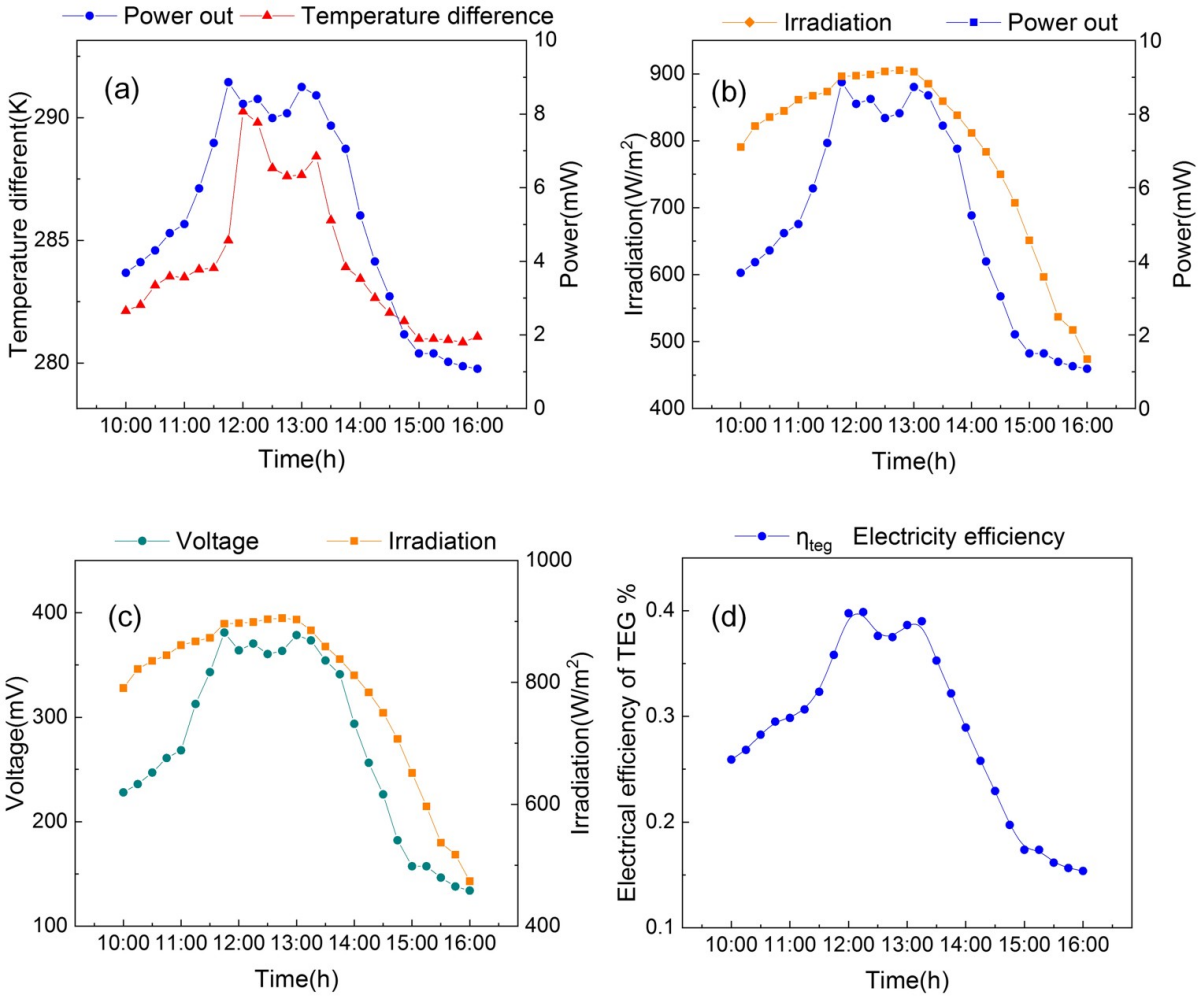
(a) Temperature difference variations and power generated by the TEG modules during the experiment. (b) Irradiation and power generated by the TEG modules during the experiment. (c) Irradiation and voltage of the TEG modules during the experiment. (d) Electrical efficiency of the TEG modules during the experiment.

The temperature difference and output power over time are shown in Fig 6(a). Both graphs exhibited the same trend. The maximum temperature difference occurred at approximately 12 pm, at 289.89 K. In Lee’s [20] floating device, the hot side of TEG modules was directly exposed to sunlight and cold side with heat sink in water, the maximum temperature difference was only 279.15 K. Ameri [23, 24] investigated the cavity with TEG numerically without Fresnel lens. The temperature difference was only 5K when the solar irradiation was around 850 W/m^2^. While the temperature difference increased by 284.89K in our system with Fresnel lens. Using Eqs (13) and (14), the load resistance (*R*_*load*_) of the floating device was determined to be 13 Ω. The output power reached its first peak at approximately 12 pm followed by second peak occurring at 1:30 pm. The experimental results indicated that Eq (14) was indeed correct and illustrated that temperature difference played a vital role in this energy conversion process.

The maximum solar irradiation occurred at approximately 1:30 pm with the change of solar irradiation angle during a day, as shown in Fig 6(b). The increased solar irradiance caused the temperature of the selective absorbing coating to increase, and then passed through the hot side of TEG modules. The output power reached an initial maximum at 8.863 mW and then decreased slightly. Compared with Omid’s alternative cavity receiver [24], whose max output power generated by TEG was only 2 mW during the afternoon, this system received fourfold as much power with the same numbers of TEG modules. Since wireless sensors network could be started with a 84 μW input power [25], the power generated by floating device was sufficient to run low-power sensors. As the solar irradiance reduced, the output power decreased, with a value of 1.08 mW at 4 pm. The voltage exhibited the same trend as the power. Traces showing the maximum voltage and solar irradiation over time are shown in Fig 6(c). The voltage reached an initial peak at 381.08 mV, and was followed by a second peak at 373.52 mV.

The hot side temperature was largely determined by the solar irradiation. The rate of water flow was the main factor that determined the temperature of the cold side. The temperature difference, power and voltage all exhibited the same trend. The maximum values of these parameters all occurred at the same time. Therefore, it is critical to increase the temperature difference between the two sides of the TEG modules using solar irradiation for high efficiency power generation.

According to Eqs (4) and (6), both thermal radiation and thermal convection reduced significantly when *T*_*ct*_ approaches to *T*_*sac*_. The cavity temperature increased as the solar irradiation increased, which was helped by the insulation. Between 11:30 am and 13:30 pm, the power output was higher because of the reduced difference between *T*_*ct*_ and *T*_*sac*_. Willars et al [9] studied the plate energy harvesting, the temperature of hot side for TEG was around 300K. In this system with cavity, the hot side temperature enhancement was about 4.3%.

The electrical efficiency of the floating device initially increased, as shown in Fig 6(d). With the increase of the solar flux, the efficiency increased and reached a maximum at 0.369%. Then it began to decrease and rose again to a second maximum of 0.322% around 1:30p.m. Finally, the electrical efficiency reduced to 0.067% as the solar irradiation decreased to a minimum at 4 pm.

**Fig 7:**
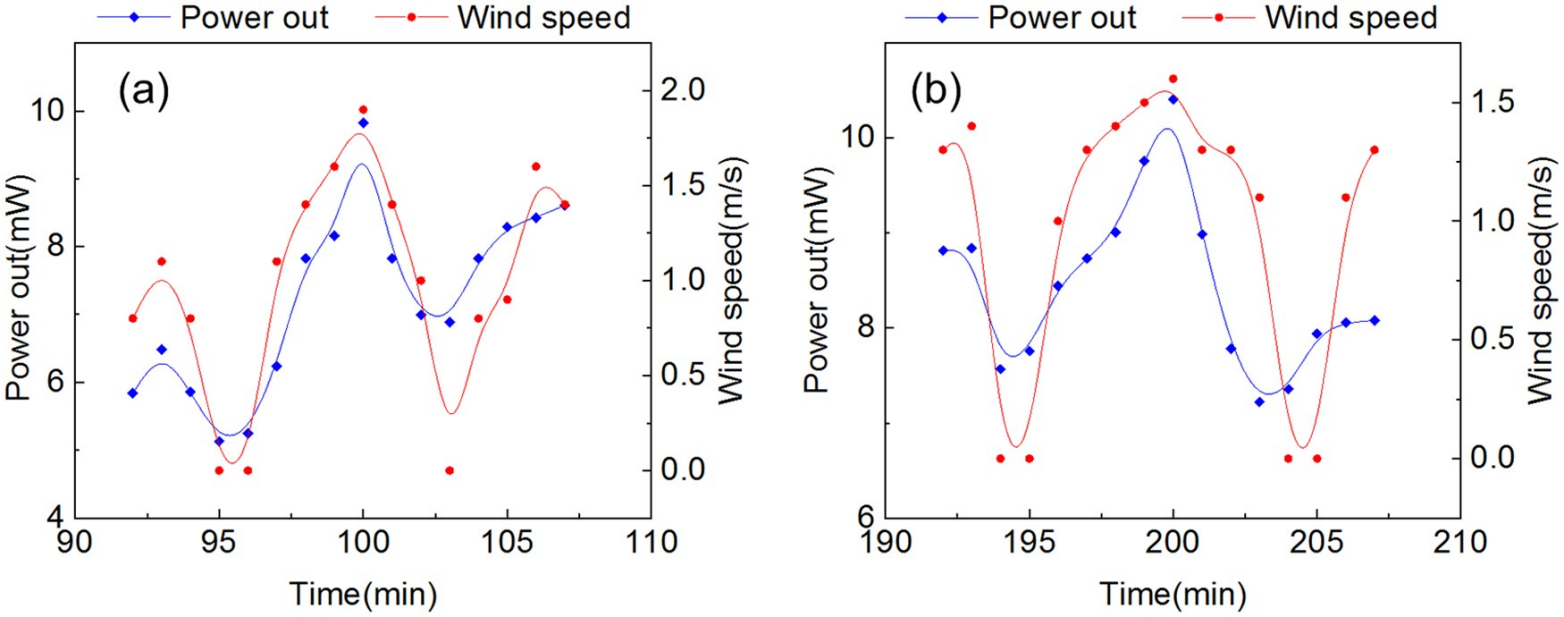
(a) Wind speed and power generated by the TEG modules from 92 min to 107 min. (b) Wind speed and power generated by the TEG modules from 192 min to 207 min.

The two peaks are worth further discussing in Fig 6. The rate of water flow on the cold side is an important factor in determining the power out, while the solar irradiation provided a large amount of heat that did not decrease significantly from 100 min to 200 min. The wind speed influenced the water flow rate. Therefore there was a close relationship between the wind speed and the power out, as shown in Fig 7(a) and 7(b). With the decrease of solar irradiance, the wind speed was not the main parameter that impacted the power out.

**Fig 8:**
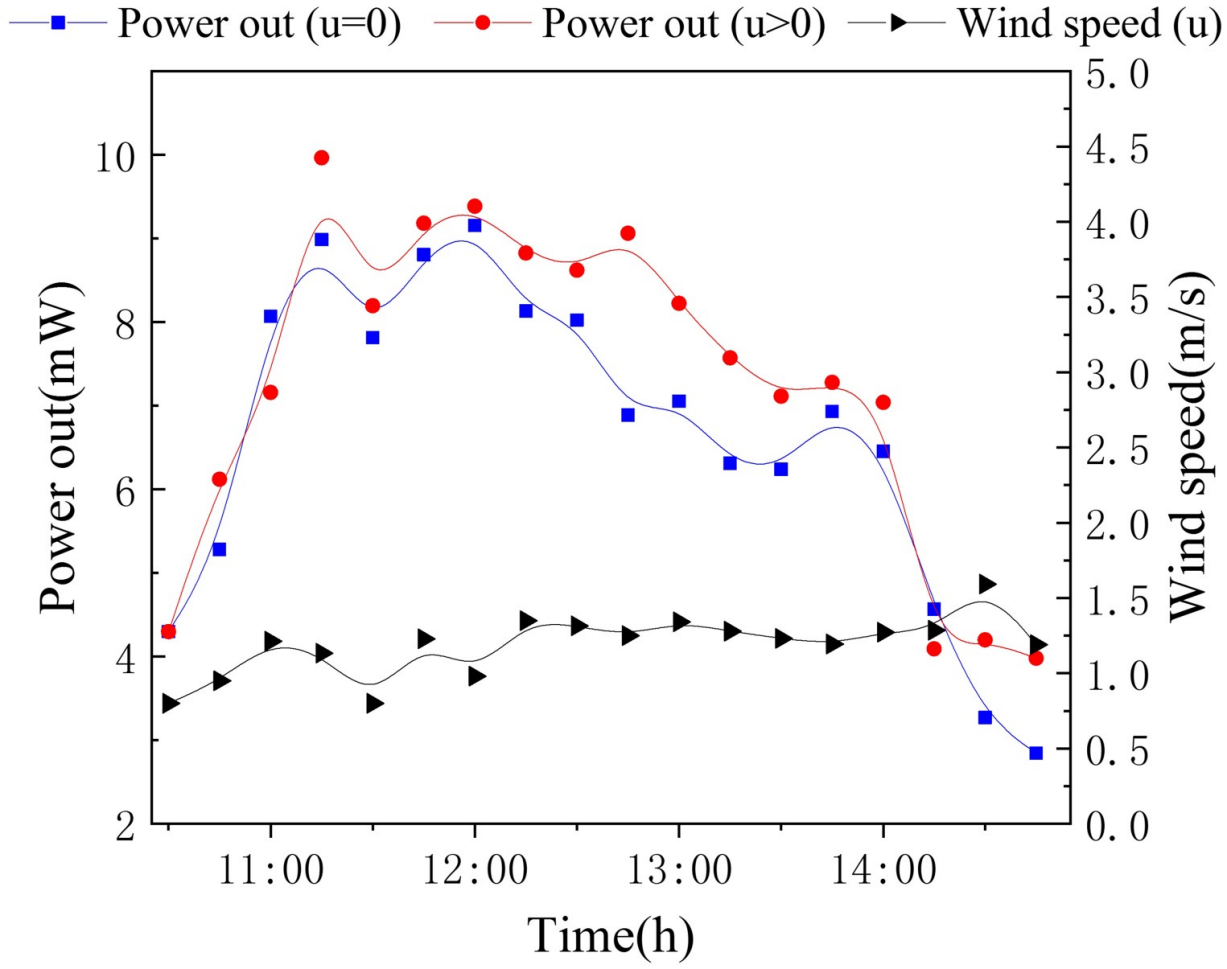
Power generated by the TEG modules with different wind speed.

In this experiment, the ranges of wind speed were 0–0.2 m/s, 0.3–1.5 m/s and 1.6–3.3 m/s, which are referred to as calm, light air and light breeze, respectively. From Fig 8, it was clear that the floating device had a higher output power when there was transient wind over the surface of water. The maximum output power was 9.96 mW and 9.16 mW for with and without wind. The output power enhancement was about 10.96% when the vertical axis rotors cached the breeze to cool the cold side of TEG. The cold side temperature was largely determined by the efficiency of heat exchanger. Compared with an alternative cavity receiver [24] without heat exchanger, the cold side temperature (*T*_*c*_) reduction was about 17K by two vertical axis rotors. The wind had a positive impact on the power out of the device because the fins drove the lower vertical rotor when the wind was stronger. This led to an increased temperature difference and resulted in more efficient power output.

Although Li et al [20] studied the influence of wind speed for heat exchangers used in atmosphere, hybrid systems that harness both breeze and water flow have not received much attention. Even if the water flow in wetlands is typically slow, any breeze over the surface of the water could be easily captured.

### Uncertainty analysis

Due to the limitation of measurement instruments, the uncertainties of indirect measurement values (*f*) must be calculated, i.e., temperature difference, maximum output power, electrical efficiency. The uncertainty data (*U*_*f*_) is defined as [26]

$$U_{f}=\sqrt{\sum_{i=1}^{n}{\left(\frac{\partial f}{\partial x_{i}}\right)}^{2}{\left(△x_{i}\right)}^{2}}$$ (21)

The precision (△*x*_*i*_) of the direct measured value (*x*) was given in Table 2

**Table 2:** Measurement accuracy of equipment.

Anempmeter (UT-363)	±(5%rdg + 0.5)
Digital multimeter (Voltmeter) DY2101	±(0.5% + 3)
Digital multimeter (Amperemeter) MT1210	±(2.0% + 5*d*)
Resistance box J2361	±1
Type K thermocouple	±1°C
TES-1333 pyrometer	±0.38W/m^2^

## Conclusions

A floating thermoelectric power generation device that concentrated solar energy for use in wetland monitoring was designed, fabricated and tested in a landscape pool under direct solar irradiation. This power generating device has great potential to remote recording a variety of information, including weather and water quality in environmental monitoring.

The influence of solar irradiation intensity, ambient temperature, the temperature of the selective absorbing coating, the cavity temperature, wind speed, maximum power output and the electrical efficiency of the TEG modules were analyzed. The main achievements and conclusions of this work were summarized as follows:

1. A novel power supply system with Fresnel lenses and a cavity structure was fabricated. It allowed efficient heating while significantly reducing thermal radiation and thermal convection. The hot side temperature increased by 4.3% in the comparison with the system without the cavity.
2. When the floating device was subjected to solar irradiation of 896.38 W/m^2^, it generated a potential difference of 381.03 mV and a power output of 8.86 mW via thermoelectric generation. The maximum temperature was 289.89 K. The power output was fourfold that of other reported systems [24] that used the same number of TEG modules.
3. Hybrid breeze and water flow could improve the power output of the system. Wind speeds below 3.3 m/s over a water surface could be used to drive the cooling system of this floating device, which increased the output power by 10.96%.
4. This novel power supply system was waterproof and could float steadily on water, which would allow it to monitor a diverse range of wetlands environments.

## Nomenclature

*A*: area of selective absorbing coating
*A*_*f*_: area of focal spot
*A*_*F*_: area of Fresnel lens
*a*_*teg*_: cross-sectional area of a p or n leg (m^2^)
*C*: concentration ratio
*C*_*p*_: Specific heat of water (kJ/(kg K))
*G*: solar radiation (W/m^2^)
*H*: height of the aluminum plate
*h*_*cov*_: coefficient of convection heat transfer (W/(m^2^ k))
*h*_*cov*−*Al*_: coefficient of convection heat transfer (W/(m^2^ k))
*h*_*rad*_: coefficient of radiation heat transfer (W/(m^2^ k))
*I*: current (A)
*k*_*Al*_: thermal conductivity of aluminum plate (W m^-1^ k^-1^)
*k*_*teg*_: thermal conductivity of TEG (W m^-1^ k^-1^)
*L*: length of aluminum plate (m)
*l*_teg_: height of TEG (m)
*n*_*teg*_: numbers of PN junction
*P*_*out*_: output power (W)
Pr: prandtl number
*Q*_con_: heat flux via conduction (W)
*Q*_cov_: heat flux via convection (W)
*Q*_in_: solar energy absorbed by selective absorbing coating (W)
*Q*_rad_: heat flux via radiation (W)
*Q*_tegh_: energy that passed in hot side of the TEG (W)
*Q*_tegl_: energy that passed out of cold side of the TEG (W)
*R*_*Al*_: thermal resistance of the aluminum plate (K W^-1^)
*R*_*load*_: load resistance (K W^-1^)
*R*_*sac*−*Al*_: thermal contact resistance between selective absorbing coating and aluminum plate (K W^-1^)
*R*_*teg*−*Al*_: thermal contact resistance between TEG and aluminum plate (K W^-1^)
*S*_*1*_: cross section area of aluminum plate (m^2^)
*S*_*2*_: total area of aluminum plate (m^2^)
*T*_*c*_: temperature of cold side of TEG (K)
*T*_*ct*_: temperature of cavity (K)
*T*_*h*_: temperature of the hot side of TEG (K)
*T*_*sac*_: temperature of selective absorbing coating surface (K)
*T*_*w*_: the temperature of water (K)
*u*: wind speed (m/s)
*u*_∞_: Water flow rate (m/s)
*v*: Dynamic viscosity (N·s/m^2^)
*α*: heat diffusivity (m^2^/s)
*α*_*b*_: absorptivity of absorbing coating
*α*_*teg*_: the relative Seebeck coefficient
*δ*: Stefan–Boltzmann constant (W m^-2^ k^-4^)
*ε*: reflectivity of absorbing coating
*η*_sys_: electrical efficiency of the system
*η*_teg_: electrical efficiency of the TEG module
*ρ*: density of water, kg/m^3^
